# Is a Proactive Approach to Controlling *Legionella* in the Environment Justified?

**DOI:** 10.17113/ftb.59.03.21.7016

**Published:** 2021-09

**Authors:** Daniela Glažar Ivče, Dobrica Rončević, Marina Šantić, Arijana Cenov, Dijana Tomić Linšak, Vladimir Mićović, Dražen Lušić, Marin Glad, Davor Ljubas, Darija Vukić Lušić

**Affiliations:** 1 Teaching Institute of Public Health of Primorje-Gorski Kotar County, Krešimirova 52a, 51000 Rijeka, Croatia; 2 Faculty of Health Studies, Viktora cara Emina 5, 51000 Rijeka, Croatia; 3 Faculty of Medicine, University of Rijeka, Braće Branchetta 20, 51000 Rijeka, Croatia; 4 Center for Advanced Computing and Modelling, University of Rijeka, Radmile Matejčić 2, 51000 Rijeka, Croatia; 5 Faculty of Mechanical Engineering and Naval Architecture, University of Zagreb, Ivana Lučića 5, Zagreb, Croatia

**Keywords:** *Legionella pneumophila*, environmental surveillance program, legionnaires' disease, preventive approach, drinking water distribution system, health and aged care facilities

## Abstract

**Research background:**

*Legionella* are Gram-negative bacteria that are ubiquitous in the natural environment. Contaminated water in man-made water systems is a potential source of transmission of legionnaires’ disease. The aim of this study is to explore the prevalence of *Legionella pneumophila* in the drinking water distribution system (DWDS) of Primorje-Gorski Kotar (PGK) County, Croatia, for the period 2013-2019, coupled with the incidence of legionnaires’ disease. A number of *L. pneumophila-*positive samples (>100 CFU/L), serogroup distribution and the degree of contamination of specific facilities (health and aged care, tourism, and sports) were assessed. Based on the obtained results, the reasoning for the implementation of a mandatory *Legionella* environmental surveillance program was assessed.

**Experimental approach:**

Sample testing for *Legionella* was carried out according to the standard method for enumeration of this bacterium. A heterotrophic plate count (HPC) and *Pseudomonas aeruginosa* number were analysed along with the basic physicochemical indicators of drinking water quality. The research period was divided into two parts, namely, the 2013-2018 period (before implementation of the prevention program, after the outbreak of legionnaires’ disease), and the year 2019 (proactive approach, no disease cases recorded).

**Results and conclusion:**

During the 7-year observation period in PGK County, an increase in the number of samples tested for *Legionella* was found. An increase in *Legionella*-positive samples (particularly pronounced during the warmer part of the year) was recorded, along with a growing trend in the number of reported legionnaires’ disease cases. In addition to hot water systems, the risk of *Legionella* colonisation also applies to cold water systems. Health and aged care facilities appear to be at highest risk. In addition to the higher proportion of positive samples and a higher degree of microbiological load in these facilities, the highest proportion of *L. pneumophila* SGs 2-14 was identified. Due to the diagnostic limitations of the applied tests, the number of legionnaires’ disease cases is underdiagnosed.

**Novelty and scientific contribution:**

The introduction of a mandatory preventive approach to monitoring *Legionella* in DWDS water samples, along with the definition of national criteria for the interpretation of the results will create the preconditions for diagnosis and adequate treatment of larger numbers of legionnaires’ disease cases.

## INTRODUCTION

Legionnaires' disease emerged in 1976 in the city of Philadelphia, PA, USA, during a convention of 4400 U.S. Army veterans (legionnaires); it affected 182 participants, and proved fatal for 34 people (*1*). Next year, McDade and his collaborators isolated the pathogen, a bacterium that was named after the legionnaires, namely *Legionella*. It belongs to the *Legionellaceae* family, and to date, at least 65 species and 70 serological groups (16 SGs *L. pneumophila*) have been identified (*1*). *L. pneumophila* is the most pathogenic species; it causes more than 90% of the infections worldwide (*2*). *L. pneumophila* SG 1 is associated with 70% of legionellosis in Europe (*3*), followed by SGs 4 and 6. *Legionella* can cause legionnaires' disease (potentially fatal atypical pneumonia) or Pontiac fever (short-term flu-like febrile illness). The average incubation time of legionnaires' disease is 2-10 days (median 6-7 days) (*1*). Individual risk factors for legionnaires’ disease include older people (90% of the patients were older than 45), immunocompromised patients, gender (men are more than twice as likely to fall ill than women), chronic lung diseases, smoking and alcoholism (*4*). According to the latest epidemiological report published by the European Centre for Disease Prevention and Control (ECDC), 8% of legionellosis cases in European Union and European Economic Area (EU/EAA) countries ended fatally (*5*).

In natural spring water, bacteria are present in small numbers, while in man-made water systems *Legionella* replicate and usually achieve high number. A key factor for their proliferation is a suitable water temperature, while the formation of a biofilm further supports their growth and reproduction, protecting them from chemical disinfectants. As *Legionella* is protected inside amoebae and biofilms, it becomes resistant to normal doses of chlorine, and thus can colonise both warm and cold water supply systems. Water aerosol inhalation is the predominant pathway of transmission, while in fewer cases it can also be transmitted by aspiration. Although it is considered that there is no human-to-human transmission, in 2014 one probable case was recorded, the only such case described to date (*6*).

The European Legionnaires' Disease Surveillance Network (ELDSNet), coordinated by ECDC (based in Stockholm, Sweden), has been monitoring this disease in Europe since 2010 (*7*). Data are collected from 28 EU member states and two EAA countries (Iceland and Norway). Two different systems are used, annual and daily monitoring. The annual monitoring system passively monitors trends in the incidence of legionellosis over time in the member states. On the other hand, daily reporting relates to the prompt notice of travel-associated legionnaires' disease (TALD) and includes non-EU/EEA countries as well. Two thirds of the reported cases in 2018 (71%) were located in four countries: France, Germany, Italy and Spain, despite the fact that the population of these countries accounts for only about 50% of the European population (*5*). The large differences in reported rates could predominantly be attributed to the various levels of development of the national monitoring systems for this disease, diagnostic methods as well as data presentation.

Around the world, numerous public health institutes and agencies have issued different guidelines for the prevention of legionellosis (*8*, *9*): U.S. CDC (U.S. Center for Disease Control and Prevention), U.S. EPA (U.S. Environmental Protection Agency), U.S. VHA (U.S. Veterans Health Administration) and U.S. OSHA (U.S. Occupational Safety and Health Administration), Australian enHealth, British PHE (Public Health England, UK), European ECDC, and Croatian Institute of Public Health. These guidelines focus primarily on building water supply systems, healthcare facilities, hotels, camps, marinas, cooling towers, cruise ships and swimming pools. Some EU member states, such as Germany, have introduced proactive strategies by imposing compulsory preventive monitoring of the presence of *Legionella* spp. in drinking water (*10*), while some have set limit values in national regulations, ranging from 100 CFU/L (Netherlands) to 1000 CFU/L (Germany, France) (*11*). In the USA, the EPA has not set a threshold for *Legionella*. The US Surface Water Treatment Rule provides treatment techniques for removal/inactivation of *Giardia* and viruses that are considered sufficient to control *Legionella* (*12*). In a document published in 2019, Public Health Ontario (Canada) outlines the benefits and concerns of implementing such programs (*13*). They draw attention to the shortcomings of routine water testing, noting that *Legionella* is ubiquitous in drinking water supply systems, scientifically based threshold levels are limited, and interpretation of test results is difficult. Then again, several public health organizations place emphasis on the usefulness of these programs, such as confirmation of the effectiveness of control measures in the prevention of legionnaires’ disease, provided it is carried out within the framework of a Water Safety Plan. Former EU Directive on the quality of water intended for human consumption (*14*) did not prescribe water testing for *Legionella*. However, a new Directive, which was published on 16 December 2020, includes this bacterium in routine, preventive monitoring (*15*). Until 2023, EU member states must implement the provisions of the new directive into their national legislation.

The aim of this study is to analyse the occurrence and load of *L. pneumophila* in water samples and the quality of drinking water provided by various types of facilities in Primorje-Gorski Kotar (PGK) County, Croatia, during the period 2013-2018. The results were compared to the results for year 2019, when preventive monitoring of the presence of *Legionella* in the water supply systems of PGK County was implemented. To the best of our knowledge, this has been done for the first time in Croatia. Based on the obtained results, the rationale for implementing a preventive program was assessed as a forthcoming obligation of the new Directive.

## MATERIALS AND METHODS

During the seven-year period, 2013-2019, 962 samples of water intended for human consumption (drinking water) were analysed for *Legionella pneumophila* in the region of Primorje-Gorski Kotar (PGK) County, Croatia. Most samples (*N*=893; 92.8%) were sampled from three types of facilities: tourism (*N*=690; 77.3%), health and aged care (*N*=126; 14.1%) and sports (*N*=77; 8.6%).

Prior to the implementation of the prevention program (2013-2018), samples were collected after the epidemiological indications, while the number of samples depended on the size of the facility. Facilities in which the presence of *Legionella* was confirmed were placed under the constant epidemiological surveillance, which was based on monthly sampling.

In 2019, during the implementation of preventive program, it was planned to take samples once, in the facilities with increased risk for the *Legionella* development. Depending on the facility size, 1-5 samples were taken. However, for the samples that returned *Legionella*-positive, regardless of *Legionella* load, sampling was repeated at the same sample site and again after the implementation of control measures (sediments and scale deposits physically cleaned, system flushing, pasteurisation and shock hyperchlorination).

Samples from tourism facilities, characterised by seasonal and all-year-round service, were sampled from: camps (*N*=331; 48.0%), hotels (*N*=330; 47.8%) and private accommodations (*N*=29; 4.2%). The health and aged care facility samples included: hospitals (*N*=72; 57.1%), retirement homes (*N*=34; 27.2%) and rehabilitation centres (*N*=20; 15.9%). In sports facilities samples were sampled mostly from sport halls (*N*=72; 93.5%) and the rest of them from stadiums (*N*=5; 6.5%). Of the analysed samples, 529 samples (59.2%) were collected from warm water distribution systems, 334 (37.4%) from cold water distribution systems, while the temperature was not recorded for 30 samples.

Samples were taken as a preflush samples without flaming of the taps in order to determine the *Legionella* colonisation of particular outlet. Water samples were collected in sterile 1-litre bottles with the addition of 1 mL of 0.0125 M sodium thiosulfate to inactivate residual chlorine. Samples were transported in ice coolers at the temperature of (5±3) °C and processed in a laboratory within the same day (exceptionally, up to 24 h, providing that they were kept cool).

### Microbiological methods

#### Detection and quantification of *Legionella* spp. in water samples

*Legionella* spp. from water samples was detected and quantified by culture in accordance with ISO 11731:2017 (*16*). During the period 2013-2016, *L. pneumophila* was isolated in accordance with the previous version of that standard (ISO 11731:1998) (*17*); however, the procedure remained unchanged. One litre of water sample was concentrated by filtration through a 0.2-μm pore size polycarbonate membrane filter, 47 mm in diameter (Pall Corporation, Ann Arbor, MI, USA). The membrane was then transferred to a screw cap sterile container with 10 mL distilled water, cut into pieces using sterile scissors to aid elution and vortexed for at least 2 min to dislodge the microorganisms from the membrane filter. A volume of 0.1 mL of the heat-treated samples was spread over a selective glycine, vancomycin, polymyxin B, cycloheximide (GVPC) agar plate (Oxoid, Thermo Fisher Scientific, Basingstoke, UK). The inoculated GVPC media were incubated for up to 10 days at (36±1) °C in a humid atmosphere with 2.5% CO_2_ and examined every 2-3 days. Presumptive colonies were subcultured on buffered charcoal yeast extract agar (BCYE) and buffered charcoal yeast extract without l-cysteine (BCYE-cys; Oxoid Thermo Fisher Scientific), or other appropriate media (*e.g*. sheep blood agar), and incubated at (36±1) °C for >2 days. The colonies that grew on the BCYE medium but failed to grow on the BCYE-cys with characteristic morphologic features were regarded as presumptive *Legionella*. Isolated colonies were confirmed using a commercially available agglutination test (DrySpot Legionella Latex Test, Oxoid, Thermo Fisher Scientific), which allows separate identification of *L. pneumophila* SG 1 and SGs 2-14. The detection limit of the described procedure was 100 CFU/L.

#### Enumeration of heterotrophic plate count

Heterotrophic plate count (HPC) was determined according to ISO 6222:1999 (*18*). A volume of 2 mL of sample (or appropriate dilution) was placed in two Petri dishes (1 mL per dish). Afterwards, 15-20 mL of molten yeast extract agar (Biolife Italiana S.r.l., Milan, Italy) were added and mixed thoroughly by rotation. After the medium has cooled (maximum 15 min), the plates were inverted. Inoculation occurred at two temperatures: one plate was incubated at (36±2) °C for (44±4) h and the other at (22±2) °C for (68±4) h. All colonies grown in the plate were counted. Results were expressed as colony forming unit (CFU) per mL.

### Detection and quantification of *Pseudomonas aeruginosa*

*Pseudomonas aeruginosa* was tested according to ISO 16266:2008 (*19*). A volume of 100 mL of water sample (or appropriate sample dilution) was filtered through a mixed cellulose ester membrane, 0.45 µm pore size and 47 mm diameter (Pall Corporation). The membrane was transferred on the Pseudomonas agar base supplemented with glycerol and CN supplement (Biolife Italiana S.r.l.). The plates were incubated at (36±2) °C for 24-48 h. All green/blue colonies were confirmed as *P. aeruginosa*. Colonies that were fluorescent under the Wood’s lamp (SPECTROLINE®, model CM-10A; Spectronics Corporation, New York, NY, USA) as well as reddish brown colonies that do not fluoresce, were counted as presumptive. All suspicious colonies were confirmed using acetamide broth test for the ability of *P. aeruginosa* to produce ammonia from acetamide (Biolife Italiana S.r.l.), oxidase test (Biolife Italiana S.r.l.) and King’s B medium that enhances the production of fluorescein (Biolife Italiana S.r.l.).

### Physicochemical parameters of water quality

Water temperature was measured in accordance with the APHA St. Method 2550 B (*20*) using alcohol thermometer with graduation intervals of 0.1 °C. Residual free chlorine was measured according to ISO 7393-2:2018 (*21*) (quantification limit of Cl_2_ 0.02 mg/L) using portable colorimeter Pocket Colorimeter™ II (Hach, Loveland, CO, USA). Electrical conductivity and pH values of the water were measured using multi-channel, modular instrument SevenExcellence (S47; Mettler Toledo, Giessen, Germany) according to ISO 7888:1985 (*22*) (quantification limit 9 µS/cm) and ISO 10523:2008 (*23*), respectively.

Turbidity was measured according to ISO 7027-1:2016 (*24*) (quantification limit 0.10 NTU) using laboratory turbidimeter 2100N IS (Hach). Permanganate index (consumption of KMnO_4_) was determined according to ISO 8467:1993 (*25*) (quantification limit of O_2_ 0.25 mg/L). Quality control was performed with resorcinol (Merck, Darmstadt, Germany) with recovery of 90-105%.

### Statistical analysis

The results are presented using descriptive statistics: relative frequency, mean value and median, standard deviation (S.D.), interquartile range (IQR) and data range) as measures of data dispersion, as well as graphically. The normality of data distribution was tested using the Kolmogorov-Smirinov test. Since data distribution did not follow the Gaussian curve, nonparametric tests (Spearman's correlation coefficient, Mann-Whitney *U* test) were performed using TIBCO Statistica v. 13.5.0 software package (*26*), at a significance level of p<0.05.

## RESULTS AND DISCUSSION

**Fig. 1:**
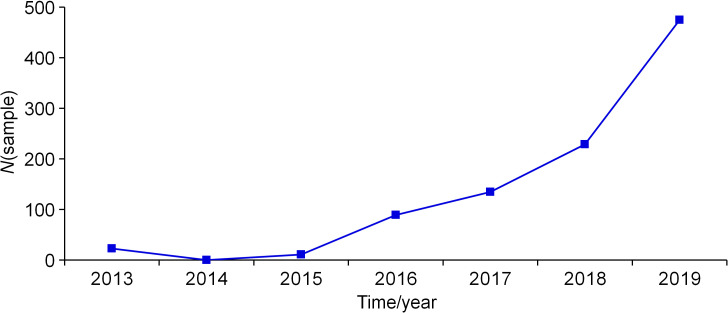
Total number of samples tested for *Legionella pneumophila* per year from 2013 to 2019. The fraction of* L. pneumophila*-positive samples was: 0.0, 0.0, 0.0, 10.1, 24.4, 22.7 and 17.9 %, respectively

In the last few decades, Croatia has been recognised as an attractive destination for tourists from all over the world, in the summer season in particular. In addition, during the last few years, health tourism has attracted different groups of people, including immunocompromised patients. This particular group is especially susceptible to all forms of respiratory diseases, including legionnaire’s disease (*27*). For these reasons, the requirements for environmental water sampling and analyses of *Legionella* have increased significantly in Primorje-Gorski Kotar (PGK) County during the 2013-2019 period. The number of tested samples grew from 0 (2014) to 475 (2019), with the share of positive samples increasing in this time interval (Fig. 1). Of the total number of samples tested for *Legionella* during the study period (*N*=962), *L. pneumophila* was confirmed in 179 samples (18.6%). The number of *L. pneumophila*-positive samples ranged from 0 (2013-2015 period) to 85 in 2019 (*N*(sample)=475), *i.e*. 17.9%.

**Fig. 2:**
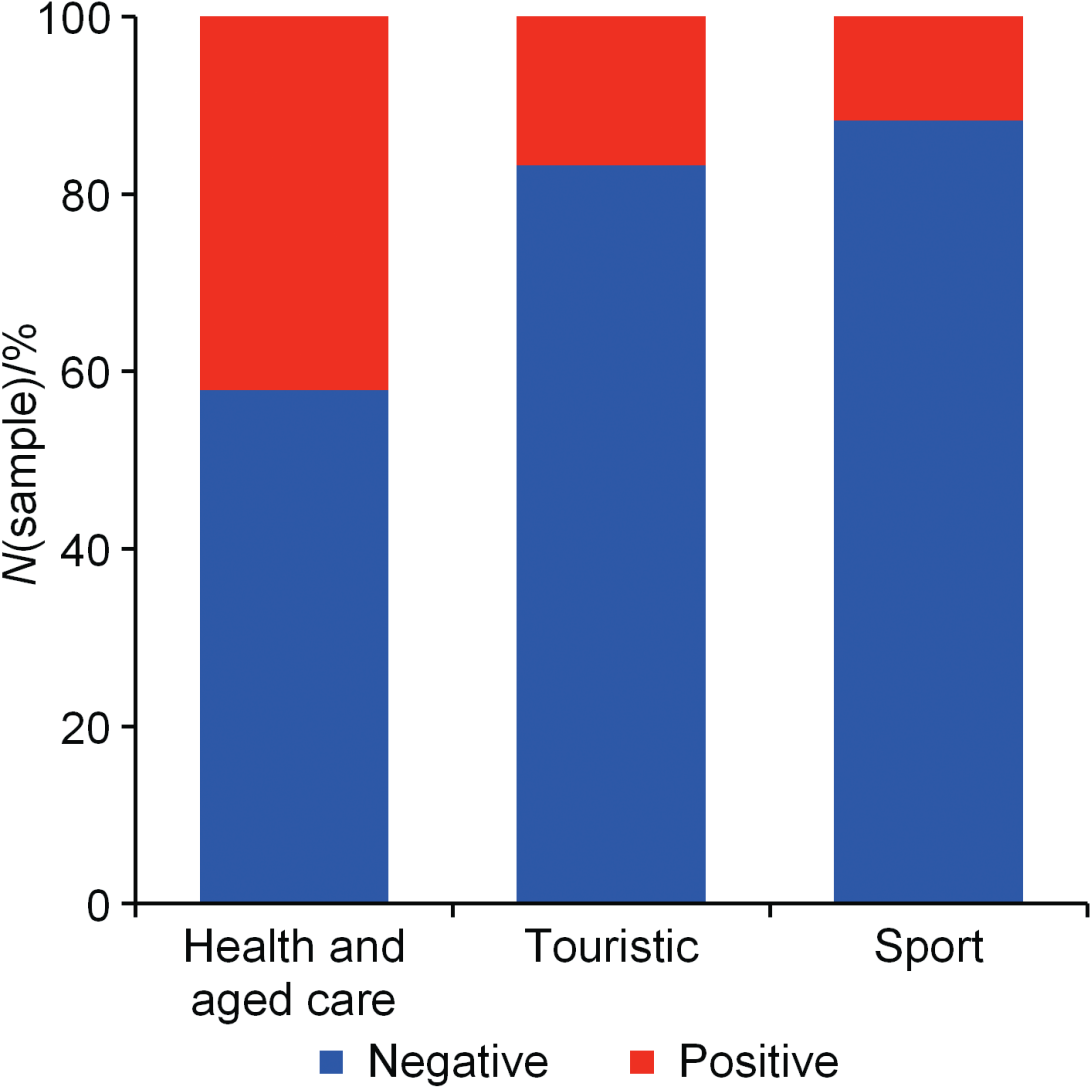
Fraction of *Legionella pneumophila*-positive samples at health and aged care, touristic and sport sampling sites: 42.1, 16.8 and 11.7%, respectively (marked red)

The largest number of samples (893 of total of 962) was taken from various types of facilities providing drinking water: tourism (*N*=690; 72%), health and aged care (*N*=126; 13%) and sports (*N*=77; 8%). Significantly fewer samples were collected from educational institutions (*N*=24; 2.5%), the municipal water distribution system (*N*=15; 1.6%), residential buildings (*N*=13; 1.4%), business offices (*N*=13; 1.4%) and service areas (*N*=4; 0.4%). Considering the total number of *L. pneumophila-*positive samples at the dominant sampling sites (tourism, health and aged care and sports facilities), the highest number was associated with tourism facilities (hotels, apartments, camps; *N*=116; 65.2%), predominantly in camps (*N*=72; 62%). Nevertheless, as regards the share of positive samples per location, health and aged care facilities, with 42.1% of *L. pneumophila-*positive samples, are at the top (Fig. 2). The European report on legionnaires’ disease for year 2015 (*28*) states that by far the largest number of *Legionella-*positive samples (in 96% of *L. pneumophila* cases) were sampled from water supply system facilities (90%), followed by cooling towers (5%) and swimming pools (3%), while only 2% was attributed to other cases.

**Fig. 3:**
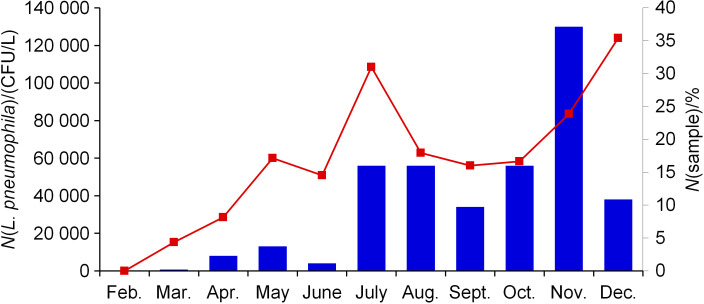
Maximum monthly load of *Legionella pneumophila* (CFU/L) from 2013 to 2019 (blue bars) and corresponding *L. pneumophila*-positive samples (marked red)

The monthly distribution of *L. pneumophila*-positive samples, along with the *Legionella* load, indicates a lower presence of *L. pneumophila* from February to June and a higher from one from July to December. The highest share of positive samples was recorded in December (35.4%), while maximum concentration was found in November (130 000 CFU/L) (Fig. 3). This noteworthy increase in the *Legionella* load during the second, warmer part of the year mostly coincides with the ECDC distribution data (*5*) of legionnaires’ disease cases per month. According to such data, 57% of legionnaires’ disease cases occur from June to October. The reason for the elevated levels of *Legionella* is attributed to higher water temperatures in the warmer months. This is especially evident in the water supply systems of the Mediterranean cities (*29*, *30*). It is also supported by a study conducted in Split-Dalmatia County (*31*), where 43.5% of positive samples were found during the July-September period. The Mann-Whitney *U* test showed that water temperatures were significantly higher from July to December than in the first half of the year (z=-2.73, p=0.006). Furthermore, during the aforementioned period, the number of tourists in holiday accommodation increased, leading to higher exposure to potentially contaminated water.

The effect of temperature on the occurrence of *Legionella* in water supply systems has been well studied (*32*, *33*). Generally, low warm water temperatures (<55 °C), as well as high cold water temperatures (>20 °C), were the most significant factors for *Legionella* growth (*34*). While temperatures of 20-50 °C are suitable for *Legionella* growth, temperatures in the range of 35-45 °C are considered as optimal (*33*). In *L. pneumophila*-positive samples, the cold water temperature was statistically significantly higher (z=3.89, p<0.001) and the warm water temperature significantly lower (z=-6.79, p<0.001) than in the *L. pneumophila*-negative samples. The median temperature in *L. pneumophila*-positive cold water samples was only 0.1 °C higher than in the samples without *L. pneumophila* (20.0 *vs* 19.9 °C). However, in *L. pneumophila*-positive warm water samples, the median temperature was 9 °C lower (42.3 *vs* 51.3 °C). Similar findings have been reported from Iran (*35*) and Greece (*32*).

**Fig. 4:**
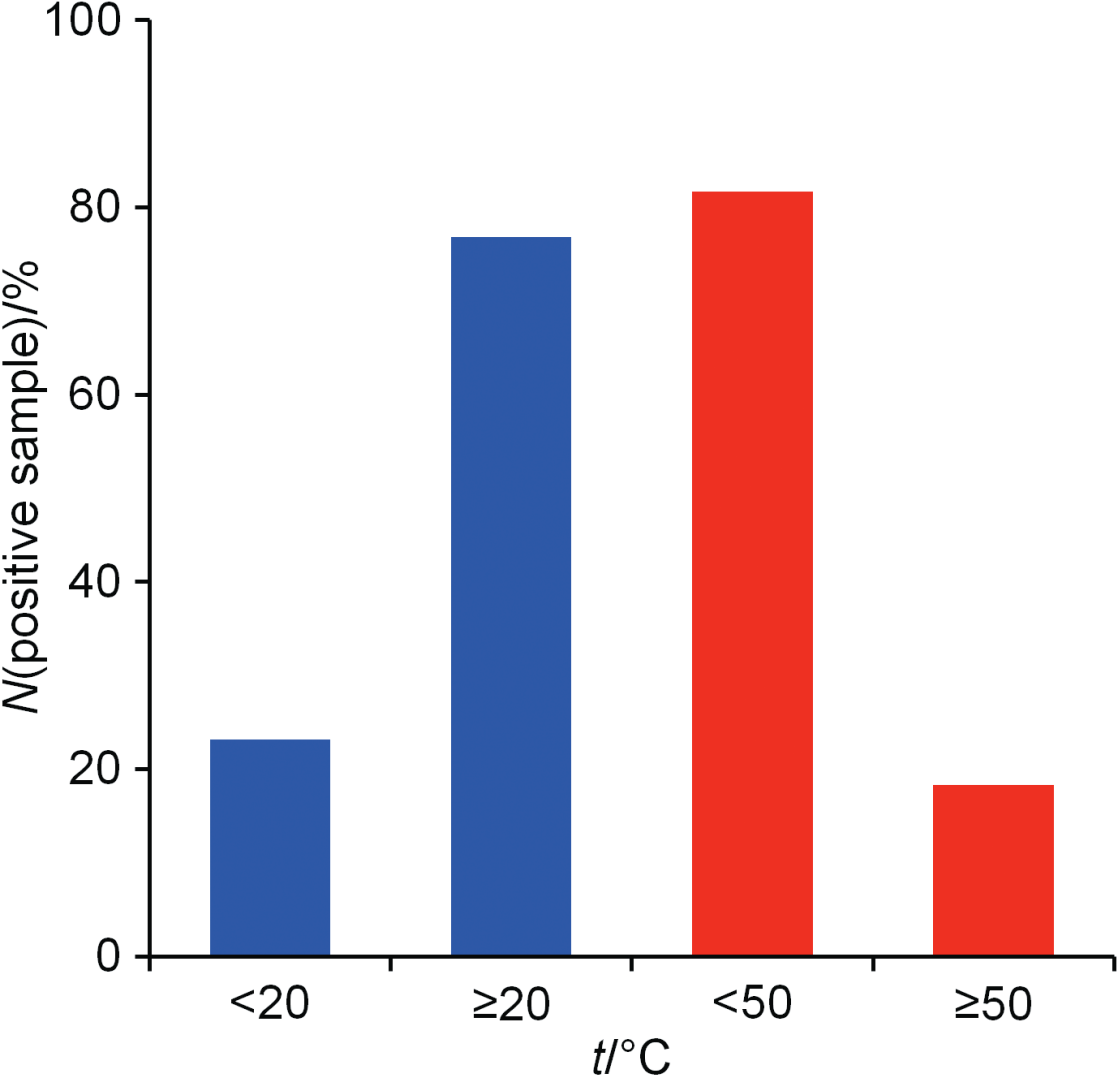
Fraction of *Legionella pneumophila*-positive samples according to the temperature range in: a) cold (blue) and b) warm (red) water distribution systems

Concerning the fraction of positive samples in cold or warm water systems, it appears that they are rather similar (20.7 and 19.6%, respectively). In 76.8% of cold water positive samples, water temperature was above 20 °C, and in 81.7% of warm water samples below 50 °C (Fig. 4).

The median concentration of *Legionella* in the warm water supply systems was slightly higher than in the cold systems (2.8 *vs* 2.0), but the difference was not statistically significant. Our findings confirmed that in PGK County, an inadequately maintained cold water supply system was equally prone to *Legionella* colonisation. The fact that in 41.6% of these samples (69 of 166, with available chlorine data) chlorine concentrations were low (≤0.02 mg/L) speaks in favour of conditions favourable to the survival of *L. pneumophila*. Residual free chlorine revealed a weak but significant negative correlation with *L. pneumophila*, heterotrophic plate count (HPC) at 37 and 22 °C (r_s_=-0.07, r_s_=-0.17, r_s_=-0.14; p<0.05, respectively), but the presence of *Pseudomonas aeruginosa* was not affected. The values of the examined physical and chemical indicators varied as follows: electrical conductivity 193-841 µS, pH=7.1-8.2, turbidity 0.15-93 NTU and consumption of potassium permanganate (expressed as O_2_) 0.23-4.1 mg/L.

**Fig. 5:**
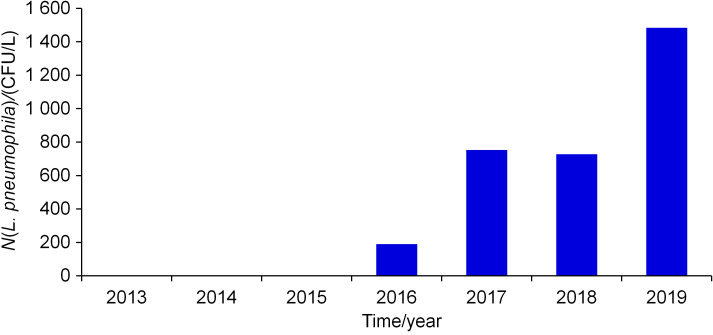
Mean annual content of *Legionella pneumophila* in the tested samples for the time period 2013-2019

Regarding the infectious dose, the load of *Legionella* in water causing disease remains unclear, but it is believed that is, for virulent strain/serotypes, rather low for immunocompromised individuals. Sikora *et al.* (*36*) state that *Legionella* numbers of 10^3^-10^5^ CFU/L can cause a sporadic form of the disease, whereas an outbreak form can be expected with values >10^5^ CFU/L (found in two study samples). The infective dose and immune status of the host are considered as crucial for the appearance of clinical signs of disease. During the observed years, the mean yearly load of *L. pneumophila* (CFU/L) in samples increased from <100 (2013-2015) to 1484 CFU/L (2019) (Fig. 5).

**Fig. 6:**
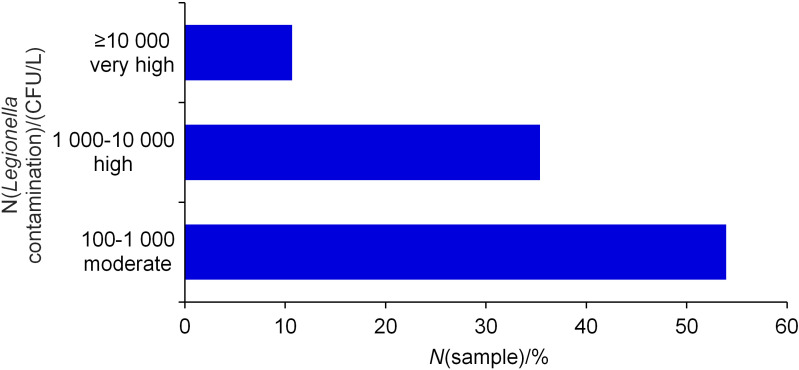
Fraction of *Legionella*-positive water samples in relation to the *Legionella* load for the time period 2013-2019

More than half of the *L. pneumophila*-positive samples (*N*=96; 53.9%) were contaminated with medium amount of *Legionella*, from 100 to 1000 CFU/L, 35.4% of the samples (*N*=63) with high amount of *Legionella*, and 10.7% (*N*=19) very high amount of *Legionella*, with more than 10 000 CFU/L (Fig. 6).

As regards the sampling locations, the lowest mean amount of *Legionella* ((670±4289) CFU/L) was recorded in tourism facilities. Higher *Legionella* loads were found in health and aged care facilities ((1911±6692) CFU/L) and sports facilities ((11258±20806) CFU/L), as a result of size, complexity and poorly designed water systems, which is also reported in other studies (*37*, *38*).

**Fig. 7:**
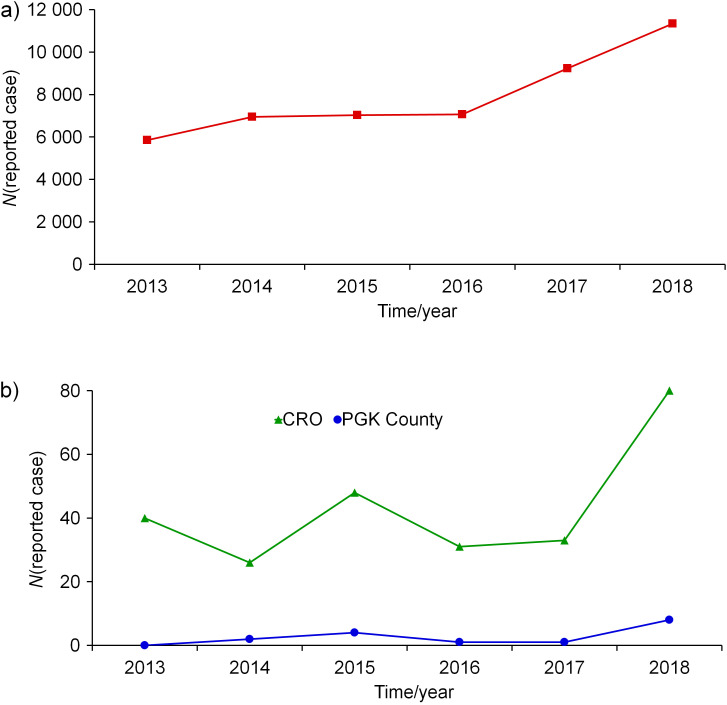
Number of reported legionnaires’ disease cases in the period 2013–2018 in: a) EU/EAA countries and b) Croatia (CRO) and Primorje-Gorski Kotar (PGK) County

Across the world and in the EU/EEA countries, including Croatia, as well as in the PGK County, the number of reported cases of legionnaires’ disease is increasing (*39*). Data provided by the US CDC reveals a 100% rise in the number of reported cases during the period 2013-2018 (from 4954 in 2013 to 9933 in 2018) (*40*, *41*). During the same period, EU/EEA countries reported a 94% increase in the number of cases, from 5851 in 2013 to 11 343 in 2018, which was the highest number ever observed. *L. pneumophila* SG 1 is the most commonly identified serogroup, accounting for 85% (*N*=909) of the cases. During the same period, in Croatia and PGK County, the number of cases was on the rise, but not continuously. The number of reported cases in Croatia ranged from 27 in 2014 to 80 in 2018, while in the PGK County it ranged from 0 to 8 (Fig. 7).

**Fig. 8:**
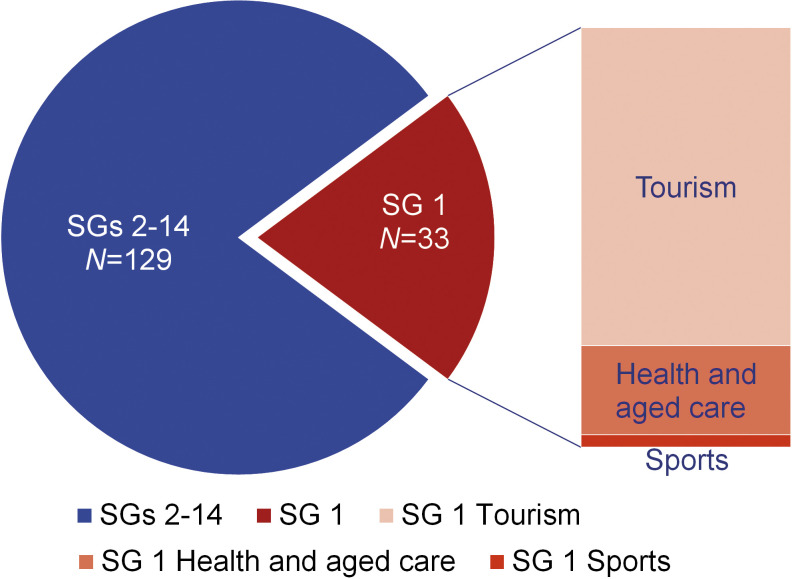
Distribution of *L. pneumophila* serogroups (SG 1 (20%) *vs* SGs 2-14 (80%)) per facility. *N*_total_=162

Of the total *Legionella* isolates in Croatia (*N*=179), the strain of 162 was identified. Four fifths of these belong to strain SGs 2-14 (80%; *N*=129). The distribution of *Legionella* serogroups differed per type of facilities. Of the total number of samples in which SG 1 was detected (*N*=33), the majority originate from tourism facilities (*N*=25; 76%) (Fig. 8). As regards water temperatures, SG 1 was present in samples with a higher measured temperature (median 43 °C) than SGs 2-14 (median 25 °C) (z=-2.90, p=0.004).

It is thought that an increase in the number of reported legionnaires’ disease cases is the consequence of several factors, from improvements to the surveillance system of the disease, an ageing population (90% of legionnaires’ disease in 2018 were found in people over the age of 45) (*5*) and climate change (*42*). Based on the above, a further increase in the legionnaires’ disease, which causes around 450 deaths annually in the EU/EEA according to the ELDSNet (*43*), can be expected. The annual notification rate of legionnaires’ disease per 100 000 inhabitants in EU/EEA countries increased by 83% during the period 2013–2018 (from 1.2 in 2013 to 2.2 in 2018) (*5*, *44*). With a number ranging from 0.6 (26 reported cases in 2014) to 1.1 (48 reported cases in 2015), Croatia reported less than one case per 100 000 inhabitants, *i.e*. less than Bulgaria, Cyprus, Finland, Greece, Hungary, Ireland, Lithuania, Poland, Romania and the United Kingdom. According to the latest report (*5*), Croatia reported 1 case per 100 000 inhabitants (43 reported cases). Six countries reported more than 3 cases per 100 000 inhabitants, namely, Slovenia (7.7 reported cases), followed by Italy (4.9), Denmark (4.6), The Netherlands (3.4), Spain (3.3) and France (3.2). The significant differences in the number of reported cases among EU/EEA countries are primarily attributed to an undersized disease monitoring and diagnosis system (*45*). Therefore, at EU level, one of the priorities should be to help countries with low notification rates improve the diagnosis and reporting of legionnaires’ disease (*5*).

### Proactive strategies

The study period was divided into two parts: (*i*) first part (2013-2018) – before preventive measures were applied, and (*ii*) second part (2019) – after the implementation of the proactive program. A similar number of samples were taken during these two periods (451 *vs* 442). The share of *L. pneumophila*-positive samples decreased during the second part of the study (2019) by only 8.7%. At first glance, this was surprising considering that samples were collected and analysed as part of the preventive measures, as opposed to the first study period when samples were taken as part of the anti-epidemic measures (only after the detection of disease cases). This might be due to the fact that in the first phase of the research, the largest number of samples (>97%) was collected at tourism facilities, while the number of samples from health and aged care and sports facilities increased significantly during implementation of the prevention program (1.8-26.7 and 1.1-16.3%, respectively). The reporting of travel-associated cases of legionnaires’ disease (TALD) through the ELDSNet is well-developed, and EU and non-EU countries with better legionnaires’ disease diagnosis capabilities are much more involved in the detection of the disease than is currently possible in Croatia. At the Teaching Institute of Public Health of Primorje-Gorski Kotar County, a *Legionella* urinary antigen test was the most common detection method for legionnaires’ disease cases. However, this test detects only *L. pneumophila* SG 1, leaving a number of cases undetected (*46*). For other species of *Legionella* and other serogroups of *L. pneumophila*, it is necessary to apply the cultivation method or PCR method, which are used increasingly for the diagnosis of legionnaires’ disease in developed countries (*47*). According to the EU report of 2018, 91% of all cases of legionnaires’ disease were confirmed by urinary antigen test (UAT) (*5*). Therefore, it can be assumed that a certain number of cases in Croatia (and in the EU, too) remain undiagnosed, especially in high-risk facilities such as health and aged care facilities, as confirmed by other studies (*38*). Moreover, Soda *et al*. (*38*) state that in such facilities, legionnaires’ disease is often caused by different species of *Legionella* or non-SG1 *L. pneumophila*. These observations are in agreement with the results of our study, according to which positive *Legionella* isolates from water plumbing system of health and aged care facilities are dominated by serogroups S2-14 (84%). Therefore, these facilities are at increased risk of legionnaires’ disease transmission (frequently old and complex systems), and on the other hand, the users of these facilities belong to a very sensitive population group (patients and elderly population). Furthermore, they are subject to a higher probability of infection and severity of the disease, while mortality climbs to as high as 40% (*27*). These facts highlight the urgency of implementing a *Legionella* prevention program at high-risk facilities (hospitals and nursing homes).

The true incidence of legionnaires’ disease in Europe remains unknown for several reasons, namely, atypical disease symptoms that make it difficult to distinguish it from other types of pneumonia; rapid administration of antibiotics without further investigation of the cause of the pneumonia; insufficient sensitivity and specificity of the applied diagnostic tests; the limitation of the most commonly used urinary test that detects only *L. pneumophila* SG 1, leaving other serogroups and species undetected; the assumption that another severe disease affecting immunocompromised patients is the cause of death, without further investigation into legionnaires’ disease; milder form of the disease; and insufficiently developed reporting system for TALD cases (*48*). The cause of non-reporting, nosocomial legionnaires’ disease in particular, may also be fear of possible court trials and financial liabilities, since the disease is considered preventable (*49*). Also, it should be noted that one of the reasons for underreported and underdiagnosed legionnaires’ disease numbers is occasional environmental testing for *Legionella* in water samples, as the data suggest that the detection of *Legionella* in distribution systems helps to increase the number of diagnosed cases (*50*).

In the second part of the investigation, the microbiological load of samples increased significantly. The share of high and very high *Legionella* contaminated water samples increased (50.0 and 15.5%, respectively) compared to the first part of the study (15.5 and 5.3%, respectively). Apart from *Legionella*, the water samples collected during the second part of the study were characterised by significantly higher HPC at 37 °C/48 h and HPC at 22 °C/72 h as well as *P. aeruginosa* (z=-4.98, p<0.001; z=-5.59, p<0.001 and z=-2.16, p<0.03, respectively). The reason for this may be the complexity and age of water supply systems, which is particularly evident in health and aged care facilities. Although *Legionella* and *P. aeruginosa* had long been considered as a cause of waterborne infections associated with health care systems (*10*, *51*, *52*), these two parameters had not been correlated. HPC at 37 °C/48 h and HPC at 22 °C/78 h showed a significant positive correlation with the *Legionella* count (r_s_=0.25 and 0.33, respectively), which was also found by Solimini *et al.* (*53*). It has previously been shown that *P. aeruginosa* may inhibit the growth of *L. pneumophila* in the aquatic environment (*54*, *55*).

The samples taken and analysed within the framework of the preventive environmental study of the presence of *L. pneumophila* in the water supply systems of the PGK County, together with the increase in the number of reported legionnaires’ disease cases point to the need for stronger surveillance and proactive actions in the control of this disease. Legionellosis requires the design of prevention programs and the definition of national guidelines and policy to prevent this disease. In Croatia, this should start with systematic environmental surveillance of the water supply systems at high-risk facilities that deliver water to vulnerable population groups, as in the Primorje-Gorski Kotar region. It is only in conjunction with the implementation of control measures that such a program will contribute to the reduction of *Legionella* in water systems and, consequently, a lower rate of legionnaires’ disease morbidity and mortality.

## CONCLUSIONS

The number of samples tested for *Legionella*, the fraction of *L. pneumophila*-positive samples, and the number of recorded legionnaires’ disease cases are increasing. The incidence of *Legionella* is higher during the warmer part of the year. As regards sampling locations, the largest number of positive samples was obtained from the water distribution systems of health and aged care facilities. These, in addition to sports facilities, are also the most microbiologically loaded. The results point to a similar prevalence of *Legionella* in the hot and cold water plumbing systems of buildings. This also indicates that inadequately maintained cold water supply systems have a similar potential for *Legionella* colonisation. Four fifths of the isolates of environmental samples were identified as *L. pneumophila* SGs 2-14, pointing to potential underdiagnosed cases, particularly in health and aged care facilities. The fraction of *L. pneumophila*-positive samples was similar during the period before (after reported legionnaires’ disease cases) and after the application of the prevention program (without the identified disease cases), which confirms the fact that a proactive approach is justified for controlling *Legionella* in the environment. The results of this research stress the need to implement a mandatory prevention program. This is especially emphasised for high-risk facilities (hospitals, nursing homes, gyms and seasonal hotels). Furthermore, national criteria should also be defined to allow interpretation of results and determine further course of action.

Our study is subject to several limitations. We performed preflushing sampling exclusively, with the priority aim of simulating the consumer exposure. However, this sampling method does not allow the assessment of the presence/load of *Legionella* in distal points of the drinking water distribution system. This is particularly pronounced for heterotrophic plate counts, a parameter that is significantly influenced by the type of water outlet and hygienic conditions. Additionally, the majority of legionnaires’ disease cases were confirmed by urinary antigen tests, which detect only the *L. pneumophila* SG 1. On the other hand, the time-consuming standard culture-based method (results may be available in up to 10 days) underestimates the number of *Legionella* in waters with high bacterial background flora or in situations when *Legionella* enter a viable but non-culturable state. Therefore, possible matching and epidemiological comparison of clinical and environmental isolates are very limited, which makes difficult tracking the source of infection. When establishing the effective *Legionella* preventive and control measures, information about the origin of the infection is the priority.
